# First Cobalt(II) Spin Crossover Compound with N_4_S_2_-Donorset

**DOI:** 10.3390/molecules25040855

**Published:** 2020-02-14

**Authors:** Fabian Fürmeyer, Danny Münzberg, Luca M. Carrella, Eva Rentschler

**Affiliations:** Department of Chemistry, Johannes Gutenberg University Mainz, 55128 Mainz, Germany; fuermeyer@uni-mainz.de (F.F.); dmuenzbe@students.uni-mainz.de (D.M.); carrella@uni-mainz.de (L.M.C.)

**Keywords:** spin crossover, iron(II), cobalt(II), N_4_S_2_-donorset, 1,3,4-thiadiazole, magnetism

## Abstract

Herein we report the synthesis and characterization of a novel bis-tridentate 1,3,4-thiadiazole ligand (**L** = 2,5-bis[(2-pyridylmethyl)thio]methyl-1,3,4-thiadiazole). Two new mononuclear complexes of the type [M^II^(**L**)_2_](ClO_4_)_2_ (with M = Fe^II^ (**C1**) and Co^II^ (**C2**)) have been synthesized, containing the new ligand (**L**). In both complexes the metal centers are coordinated by an N_4_S_2_-donorset and each of the two ligands is donating to the metal ion with just one of the tridentate pockets. The iron(II) complex (**C1**) is in the low spin [LS] state below room temperature and shows an increase in the magnetic moment only above 300 K. In contrast, the cobalt(II) complex (**C2**) shows a gradual spin crossover (SCO) with *T*_1/2_ = 175 K. To our knowledge, this is the first cobalt(II) SCO complex with an N_4_S_2_-coordination.

## 1. Introduction

Switching metal complexes between two different electronic states, high spin [HS] and low spin [LS], by external stimuli such as temperature, light irradiation or pressure is known as spin crossover (SCO). Due to the molecular bistability and the associated change in the optical and magnetic properties upon switching, these compounds can possibly be used in memory storages, displays and sensors [1,2,3,4,5,6,7,8]. However, abruptness of the property changes and the occurrence of a thermal hysteresis is necessary for future applications. Both depend on the cooperative interactions between the metal centers in the solid state. ‘Intermolecularly’, the cooperativity can be enhanced by hydrogen bonding or *π*-*π*-stacking interactions between the complexes. ‘Intramolecularly’, in polynuclear complexes, the spin-bearing metal centers can be bridged via organic ligands, leading to close proximity and a stronger communication of these metal centers [5,9,10,11,12,13]. Although SCO coordination polymers often show large thermal hysteresis [14,15], research on discrete polynuclear, and in particular in dinuclear SCO systems recently increased because the latter have better reproducibility and easier characterization. The dimeric structural motif as the simplest and smallest model for investigating intramolecular cooperative interactions also offers potential access to three states ([HS-HS], [HS-LS] and [LS-LS]) [4,16,17]. However, the design of ligands that simultaneously act as a bridge and induce a suitable ligand field is a difficult task.

Our group recently reported on the synthesis and characterization of symmetrical dinuclear iron(II) compounds with bridging ligands based on the 1,3,4-oxadiazole as well as on the 1,3,4-thiadiazole backbone [18,19,20]. For the thiadiazole-based ligand 2,5-bis[(2-pyridylmethyl)amino]methyl-1,3,4-thiadiazole with pyridyl donor sidearms, the complexes are in the [LS-LS] state at low temperatures and show a gradual but incomplete spin crossover only above room temperature [19]. Inspired by the fruitful work of *S. Brooker* et al. [21], we herein report the modification of the previous reported ligand [19] by replacing the amino for thioether linkages. The longer C-S bonds compared to the C-N bonds give greater flexibility of the ligand, and thus, should enable the population of the [HS-LS] and/or the [HS-HS] state at elevated temperatures. However, rather than obtaining the dimeric [Fe_2_(*µ*-**L**)_2_]^4+^ complex cation, we exclusively isolated two new mononuclear complexes [M^II^(**L**)_2_](ClO)_4_ (with M = Fe^II^ (**C1**) and Co^II^ (**C2**) and **L** = 2,5-bis[(2-pyridylmethyl)thio]methyl-1,3,4-thiadiazole). While the iron(II) complex (**C1**) remains in the [LS] state as well, the cobalt(II) complex shows a gradual SCO.

## 2. Results and Discussions

### 2.1. Synthesis

**Scheme 1:**
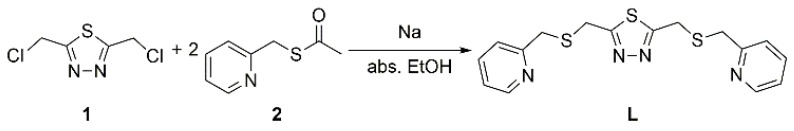
Synthesis of the Ligand (**L**) starting from 2,5-bis(chloromethyl)-1,3,4-thiadiazole (**1**).

The synthesis of the ligand is presented in Scheme 1. We have previously reported the preparation of 2,5-bis(chloromethyl)-1,3,4-thiadiazole (**1**) [19]. Thioacetic acid S-pyridine-2-ylmethyl ester (**2**) was synthesized as described in literature [21]. Finally, the ligand (**L** = 2,5-bis[(2-pyridylmethyl)thio]methyl-1,3,4-thiadiazole) was obtained according to [21] by treating **2** with sodium ethanolate and thereafter reacting with **1** in a nucleophilic substitution.

The iron(II) and cobalt(II) complexes, [M^II^(**L**)_2_](ClO_4_)_2_, have been synthesized in a stochiometric reaction of the ligand (**L**) with the corresponding perchlorate salt (Fe(ClO_4_)_4_ × xH_2_O and Co(ClO_4_)_2_ × 6H_2_O) in methanol. The compounds were obtained as single crystals (**C1** and **C2**) suitable for X-ray diffraction experiments via slow evaporation of the complex solutions. The iron(II) complex reaction was performed under nitrogen atmosphere and by using absolute methanol. The dried complexes are stable to air, no oxidation was observed.

### 2.2. Variable Temperature Magnetic Susceptibility Measurements

**Figure 1:**
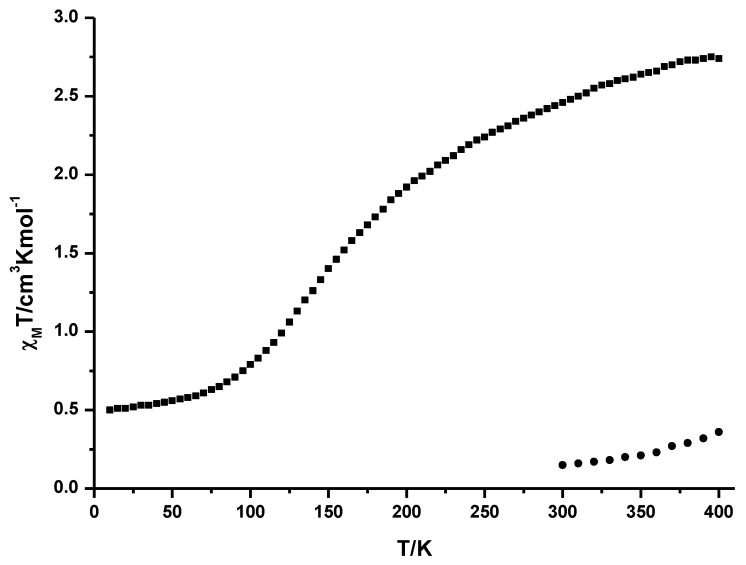
*χ_Μ_T* vs. *T* data for the compounds **C1** (dots) and **C2** (squares). The Data is given per molecule.

Variable temperature magnetic susceptibility measurements were carried on dried samples in the temperature range of 300–400 K for **C1** and of 10–400 K for **C2** in an applied magnetic field of 1000 Oe (0.1 T) and with a scan rate of 1.5 K/min. The temperature-dependent magnetic susceptibility date of the samples **C1** and **C2** are shown in Figure 1. **C1** shows a *χ_Μ_T* value of 0.15 cm^3^Kmol^−1^ at 300 K accounting for a diamagnetic iron(II) ion in the [LS] state. Also, the structural data obtained by X-ray crystallography at 173 K (described below) confirms an LS state of the iron(II) indicating that no spin crossover occurs until 300 K. Raising the temperature to 400 K, the *χ_Μ_T* value slightly increases to 0.36 cm^3^Kmol^−1^. Although this rise is no evidence of a spin crossover, it is at least a strong indication. The diamagnetic nature of **C1** at room temperature is further confirmed by the ^1^H-NMR spectra of the complex, shown along with that of the ligand in Figure S3 in the supporting information.

At low temperature, compound **C2** shows a *χ_Μ_T* value of 0.47 cm^3^Kmol^−1^, which accounts for a cobalt(II) ion in the [LS] state in accordance with the single X-ray structure analysis at 120 K. With increasing temperature, the *χ_Μ_T* product remains almost constant until 100 K, then raises up to 2.20 cm^3^Kmol^−1^ at 250 K. This is explained by a gradual spin transition of the complex from [LS] to [HS] with a transition temperature *T_1/2_* of 175 K. No magnetic hysteresis is observed. In fact, when using a cooling/heating rate of 1.5 K/min, the *χ_Μ_T* vs. *T* curves for the heating or cooling mode cannot be distinguished. The *χ_Μ_T* values for [LS] and [HS] cobalt(II) are slightly higher than the calculated ones ([LS] = 0.38 cm^3^Kmol^−1^ and [HS] = 1.88 cm^3^Kmol^−1^) using the spin-only formalism. This is expected because the spin-only formalism does not take into account orbit angular momentum contribution.

It is known from literature that cobalt(II) complexes with N-donor ligands, which form with iron(II) only [LS] complexes, might show SCO and is well studied for terpyridine complexes [2,4,22]. However, to the best of our knowledge, the cobalt(II) complex reported here is the only one showing this phenomena with Co(II) in a N_4_S_2_ coordination.

### 2.3. Crystal Structures

**Figure 2:**
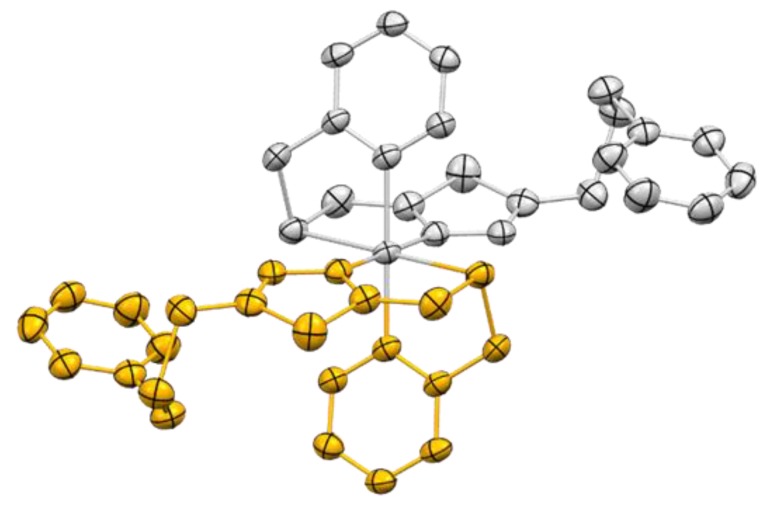
Sketch of the pseudo centrosymmetry of the complex cation representative for all three structures of **C1** and **C2**.

The complex [Fe^II^(**L**)_2_](ClO_4_)_2_ (**C1**) crystallizes in the monoclinic space group P2_1_/c at 173 K. The crystal structure of complex [Co^II^(**L**)_2_](ClO_4_)_2_ (**C2**) was measured at two different temperatures (120 K and 250 K) to confirm the spin crossover phenomenon. For both temperatures, the monoclinic space group is P2_1_/c. In all three structures, **C1** (@173 K) and **C2** (@120 K) and **C2** (@250 K) the complex cation consists of one metal ion and two ligand molecules, showing pseudo centrosymmetry as sketched in Figure 2. Each ligand contributes with one of the tridentate N_2_S binding pockets to the N_4_S_2_ octahedral coordination sphere. The second potentially donating binding pocket is not coordinating.

**Figure 3:**
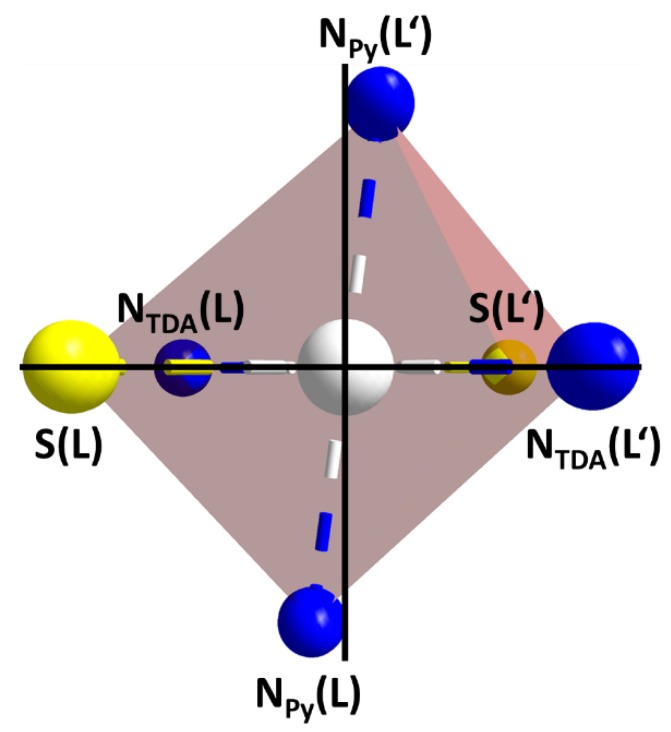
Coordination octahedron for the metal(II) ion of **C1** and **C2**.

For all complex cations (**C1** and **C2** at both temperatures), the *cis*-angles within the donor atoms of one ligand (N_TDA_(**L**,**L’**)-M-N_Py_(**L**,**L’**), N_TDA_(**L**,**L’**)-M-S(**L**,**L’**) and N_Py_(**L**,**L’**)-M-S(**L**,**L’**), Figure 3) are ranging from 83° to 85°, while the *cis*-angles between the donor atoms of the different ligands (N_TDA_(**L**)-M-N_Py_(**L’**), N_TDA_(**L**)-M-S(**L’**) and N_Py_(**L**)-M-S(**L’**) and vice versa) are ranging from 95° to 97°. This results in *trans*-angles (N_TDA_(**L**)-M-N_TDA_(**L’**), N_Py_(**L**)-M-N_Py_(**L’**) and S(**L**)-M-S(**L’**)) of almost 180° and in a slightly distorted octahedral coordination for the metal centers, in which the axis N_Py_(**L**)-M-N_Py_(**L’**) is inclined around 5–6° from the ideal geometry (black lines) towards the ligands due to the strain within the ligand backbone.

The crystal structures further compromise two perchlorate anions to counterbalance the charge. All the complexes crystallize without any solvent molecules, which allows to investigate dried crystalline samples.

The average Fe-N bond length of 1.990 Å and Fe-S bond length of 2.264 Å in **C1** (@173 K) are in accordance with those reported in literature and account for an iron(II) ion in the [LS] state [23,24,25,26,27,28,29]. Figure S7 shows the crystal structure/asymmetric unit of **C1** (@173 K). Detailed information on bond lengths and angles for **C1** and **C2** are summarized in Table 1.

**Figure 4:**
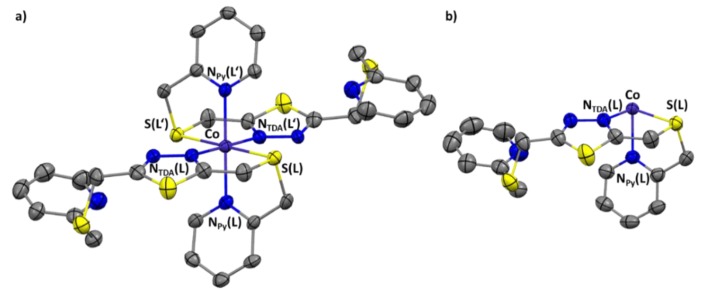
Crystal structures of the complex cations of **C2** (asymmetric unit) with thermal ellipsoids at (**a**) 120 K and (**b**) 250 K. Hydrogens, solvent molecules and counter ions have been omitted for clarity. Color code: Co is dark blue, N blue, S yellow, and C grey.

For **C2** (@250 K), the average Co-N bond length of 2.066 Å, as well as the average Co-S bond length of 2.479 Å indicate a [HS] cobalt(II) ion [30,31,32,33,34,35,36]. Cooling to 120 K results in a spin crossover from the [HS] to the [LS] state for the cobalt(II) center as shown by magnetic data. The average Co-N distance decreases to from 2.066 Å to 2.002 Å, which is in accordance with literature [37,38,39]. The shortening of the Co-N bond is explained by the decrease of electron density in the antibonding d-orbitals from t_2g_^5^e_g_*^2^ in the [HS] state to t_2g_^6^e_g_*^1^ in the [LS] state. Notably, the average Co-S distance remains about the same (2.479 Å @250 K and 2.472 Å @120 K) upon changing the electronic state of the cobalt(II) ion. This is explained by the Jahn-Teller distortion expected for a d^7^-Co(II) ion in [LS] state, with four short Co-N bonds in equatorial plane and two long ‘axial’ Co-S bonds. Uponcooling down the transition from [HS] to [LS] also affects the counter ion. Ordering of one of the perchlorate anions in the crystal structure results in a phase change. While at 250 K only half of the complex is in the asymmetric unit, the entire complex cation is found at 120 K. This is accompanied by a doubling of the cell volume from 1990 Å^3^ (@250 K) to 3874 Å^3^ (@120 K) (see Figure 4 and Figures S8 and S9 in ESI).

**Figure 5:**
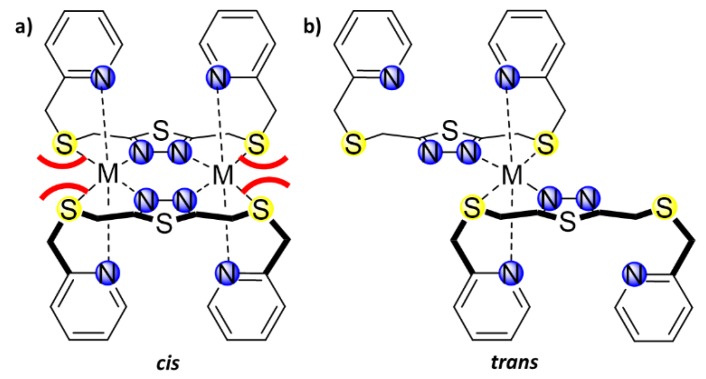
Schematic representation of the two coordination modes for the sulphur donor atoms. (**a**) The sulphur donor atoms are coordinating *cis* to each other, which is necessary to obtain dinuclear complexes. (**b**) The sulphur donor atoms are coordinating *trans* to each other, as described for the mononuclear complexes herein.

When comparing our findings with the dinuclear structures obtained by *S. Brooker* et al. [21], the question arises, why the use of our new ligand (**L**) results in mononuclear complexes? In the dimeric complexes of *S. Brooker* et al. two iron(II) ions are coordinated by two ligand molecules, thus each iron(II) center has a N_4_S_2_ coordination sphere, and the two sulphur donor atoms are coordinating *cis* to each other as depicted in Figure 5a. Changing the 1,3,4-triazole to the 1,3,4-thiadiazole as the backbone in the thioether-linked ligand leads to a different strain and to closer proximity of the sulphur donor atoms in the *cis* coordination, which is highly unfavorable. Hence, we exclusively obtained mononuclear complexes in which the sulphur donor atoms are coordinating *trans* to each other (Figure 5b). Similar findings were previously reported for iron(II) complexes with 1,3,4-triazole or 1,3,4-thiadiazole bridging ligands with an amino- rather than a thioether-linker group. Here, changing from the 1,3,4-triazole to the 1,3,4-thiadiazole backbone results in a larger angle between the intraligand donor atoms and the amine donor atoms of the two facing ligands, which are in closer proximity compared to the ones in the 1,3,4-triazole [19].

## 3. Materials and Methods

### 3.1. General Methods and Materials

All chemicals were purchased from Alfa Aesar, Deutero, Fisher Chemicals, TCI, Sigma-Aldrich and Acros Organics and used without further purification. Absolute solvents were dried according to known procedures and used freshly distilled [40]. The NMR spectra were recorded at room temperature with a Bruker Avance DSX 400 and analyzed with the program MestReNova [41]. Magnetic susceptibility measurements were performed on a Quantum Design SQUID magnetometer MPMSXL in a temperature range between 10–400 K with an applied field of 1 kOe. ESI and FD mass spectra as well as elemental analysis (C,H and N) were measured at the microanalytical laboratories of the Johannes Gutenberg University Mainz. X-ray diffraction data were collected at 173 K with STOE STADIVARI and at 120 K with a STOE IPDS 2T at the Johannes Gutenberg University Mainz. The structures were solved with ShelXT [42] and refined with ShelXL [43] implemented in the program Olex2 [44]. **Caution!** The prepared perchlorate complexes are potentially explosive. Even though no explosions occurred, only small amounts should be prepared and handled with care.

### 3.2. Ligand Synthesis

2,5-Bis(chloromethyl)-1,3,4-thiadiazole (**1**) and thioacetic acid S-pyridine-2-ylmethyl ester (**2**) were prepared as described in literature [19,21]. The ligand (**L** = 2,5-bis[(2-pyridylmethyl)thio]methyl-1,3,4-thiadiazole) was synthesized based on similar nucleophilic substitution to that found in literature [21].

#### 2,5-Bis[(2-pyridylmethyl)thio]methyl-1,3,4-thiadiazole (**L**)

Sodium (1.17 g, 51.0 mmol, 8.5 equiv.) was dissolved in ethanol (100 mL) at 0 °C and under nitrogen atmosphere. Thioacetic acid S-pyridine-2-ylmethyl ester (**2**) (2.11 g, 12.6 mmol, 2.1 equiv.) in ethanol (25 mL) was added, and the resulting brown solution was stirred at 0 °C for 30 min. Afterwards, 2,5-bis(chloromethyl)-1,3,4-thiadiazole (**1**) (1.10 g, 6.0 mmol, 1.0 equiv.) in ethanol (25 mL) was added at room temperature and the orange suspension was stirred for 17 h. The reaction mixture was poured into water (150 mL) and ethanol was removed under reduced pressure. The resulting suspension was extracted three times with dichloromethane (50 mL). The combined organic extracts were washed with brine (30 mL) and dried over magnesium sulfate. The solvent was removed under reduced pressure and the resulting crude product was purified by column chromatography (SiO_2_, dichloromethane/methanol 49:1) to give the pure product as brown oil. Yield: 1.54 g (4.28 mmol, 71.4%). ^1^H-NMR (400 MHz, CDCl_3_, 25 °C): *δ* = 8.57–8.51 (m, 2H, *H*-6, py), 7.67–7.54 (m, 2H, *H*-4, py), 7.32–7.29 (m, 2H, *H*-3, py), 7.19–7.05 (m, 2H, *H*-5, py), 4.01 (s, 4H, C*H*_2_, tda), 3.84 (s, 4H, C*H*_2_, py) ppm. ^13^C-NMR (100 MHz, CDCl_3_, 25 °C): *δ* = 170.7 (*C*, tda), 157.3 (*C*-1, py), 149.9 (*C*-6, py), 136.8 (*C*-4, py), 123.5 (*C*-3, py), 122.3 (*C*-5, py), 37.8 (*C*H_2_, py), 29.5 (*C*H_2_, tda) ppm. ESI-MS (MeOH): *m/z* (%) = 361.06 (96) [(M+H)^+^], 383.04 (32) [(M+Na)^+^], 743.10 (100) [(2M+Na)^+^]. Elemental analysis (C_16_H_16_N_4_S_3_): calcd. C 53.30, H 4.47, N 15.54; found C 52.83, H 4.58, N 15.78.

### 3.3. Complex Synthesis

To a yellow solution of the ligand (**L**) (0.1 mmol) in methanol (3 mL), an almost colorless solution of the corresponding metal(II) salt [0.1 mmol, Fe(ClO_4_)_2_ × xH_2_O or Co(ClO_4_)_2_ × 6H_2_O] in methanol (3 mL) was added. Slow evaporation at room temperature of the obtained orange solutions resulted in the formation of crystals suitable for X-ray diffraction after several hours. The iron(II) complex was prepared under nitrogen atmosphere and by using dried solvents.

#### 3.3.1. [Fe^II^(**L**)_2_](ClO_4_)_2_ (**C1**)

Fe(ClO_4_)_2_ × xH_2_O (27 mg) and 2,5-bis[(2-pyridylmethyl)thio]methyl-1,3,4-thiadiazole (**L**, 36 mg) were used to obtain **C1** (38 mg, 77.9%) as dark brown crystals suitable for X-ray diffraction. C_32_H_32_Cl_2_FeN_8_O_8_S_6_ [Fe^II^(**L**)_2_](ClO_4_)_2_ (975.76): calc. C 39.39, H 3.31, N 11.48; found (after drying in vacuo) C 38.96, H 3.03, N 11.28.

#### 3.3.2. [Co^II^(**L**)_2_](ClO_4_)_2_ (**C2**)

Co(ClO_4_)_2_ × 6H_2_O (36 mg) and 2,5-bis[(2-pyridylmethyl)thio]methyl-1,3,4-thiadiazole (**L**, 37 mg) were used to obtain **C2** (48 mg, 98.1%) as orange crystals suitable for X-ray diffraction. C_32_H_32_Cl_2_CoN_8_O_8_S_6_ [Co^II^(**L**)_2_](ClO_4_)_2_ (978.85): calc. C 39.27, H 3.30, N 11.45; found (after drying in vacuo) C 39.00, H 3.21, N 11.34.

## 4. Conclusions

In conclusion, using the novel bis-tridentate 1,3,4-thiadiazole ligand (**L** = 2,5-bis[(2-pyridylmethyl)thio]methyl-1,3,4-thiadiazole), we were able to synthesize and characterize two new complexes [M^II^(**L**)_2_](ClO_4_)_2_ (with M = Fe^II^ (**C1**) and Co^II^ (**C2**)). Due to the fact that mononuclear complexes were obtained, rather than the expected dinuclear ones, we assume this is due to the fact that the sulphur donor atoms of the thioether linkages are large compared to the nitrogen donor atoms of the amino linkages reported by Herold [19]. Thus, the *cis*-coordination of two sulphur donor atoms is unfavorable. The magnetic data of the mononuclear compound together with the single crystal X-ray structure analysis reveal a [LS] state for the iron(II) complex (**C1**) until 400 K. The cobalt(II) compound (**C2**) shows a gradual SCO between 100 K and 250 K from [LS] to [HS] state with a transition temperature *T_1/2_* of 175 K. To our knowledge, this is the first cobalt(II) complex with a N_4_S_2_ coordination environment, showing SCO behavior, that has been reported.

## Figures and Tables

**Table 1:** Selected bond lengths [Å] and bond angles [°] for the compounds [Fe^II^(**L**)_2_](ClO_4_)_2_ (**C1**) and [Co^II^(**L**)_2_](ClO_4_)_2_ (**C2**).

Selected Parameters [a]	C1 (@173 K)	C2 (@120 K)	C2 (@250 K)
M-N_TDA_(**L**)	1.967(1)	1.979(4)	2.057(2)
M-N_Py_(**L**)	2.014(1)	2.019(4)	2.075(2)
M-N_TDA_(**L′**)	1.964(1)	1.981(4)	
M-N_Py_(**L′**)	2.016(1)	2.027(4)	
M-S(**L**)	2.263(1)	2.472(2)	2.479(1)
M-S(**L′**)	2.264(1)	2.470(2)	
*cis* N_TDA_-M-N_Py_	84.1–95.9	84.6–95.6	85.1, 94.1
*cis* N_TDA_-M-S	85.3–94.8	82.9–97.4	83.8, 96.2
*cis* N_Py_-M-S	84.8–95.2	83.2–96.7	82.8, 97.2
av. *trans* X-M-X	179.8	179.6	180.0

[a] N_TDA_ = N donor atom on thiadiazole; N_Py_ = N donor atom pyridine; X = S, N_TDA_ or N_Py_ donor atom.

## Supplementary Materials

The following are available online at https://www.mdpi.com/1420-3049/25/4/855/s1, Figure S1: ^1^H-NMR of 2,5-bis[(2-pyridylmethyl)thio]methyl-1,3,4-thiadiazole (L). Figure S2: ^13^C-NMR of 2,5-bis[(2-pyridineylmethyl)thio]methyl-1,3,4-thiadiazole (L). Figure S3: Comparison of the ^1^H-spectra of a) [Fe^II^(L)_2_](ClO_4_)_2_ (C1) and b) 2,5-Bis{[(pyridine-2-ylmethyl)-thio]-methyl}-1,3,4-thiadiazole (L). Figure S4: Mass spectrum of 2,5-bis[(2-pyridylmethyl)thio]methyl-1,3,4-thiadiazole (L). Figure S5: IR spectrum of dried [Fe^II^(L)_2_](ClO_4_)_2_ (C1). Figure S6: IR spectrum of dried [Co^II^(L)_2_](ClO_4_)_2_ (C2). Figure S7: Molecular structure of [Fe^II^(L)_2_](ClO_4_)_2_ (C1) with thermal ellipsoids at 173 K. b) Asymmetric unit without hydrogens, solvent molecules and counter ions. Color code: Fe dark red, N blue, S yellow, C grey, H light grey, Cl green and O red. Figure S8: Molecular structure of [Co^II^(L)_2_](ClO_4_)_2_ (C2) with thermal ellipsoids at 120 K. Color code: Co dark blue, N blue, S yellow, C grey, H white, Cl green and O red. Figure S9: Molecular structure of [Co^II^(L)_2_](ClO_4_)_2_ (C2) with thermal ellipsoids at 250 K. Color code: Co dark blue, N blue, S yellow, C grey, H white, Cl green and O red. Table S1: Crystallographic parameters for the discussed crystal structures of C1 and C2. CCDC 1980403 for **C1** (@173 K), 1980401 for **C2** (@120 K), 1980402 for **C2** (@250 K) contain the supplementary crystallographic data for this paper. These data can be obtained free of charge from The Cambridge Crystallographic Data Centre via www.ccdc.cam.ac.uk/data_request/cif.
