# Supplementary material for: First Cobalt(II) Spin Crossover Compound with N4S2-Donorset
Source: Molecules. 2020 Feb 14;25(4):855. doi: 10.3390/molecules25040855 (PMC7070910; doi:10.3390/molecules25040855)
Supplement: Supplementary file 1 [file molecules-25-00855-s001.pdf]

Supplementary files

First cobalt(II) spin crossover compound with N_4S_2-Donorset

Fabian Fürmeyer [1], Danny Münzberg [1], Luca M. Carrella [1] and Eva Rentschler [1,*]

[1] Department of Chemistry, Johannes Gutenberg University Mainz, 55128 Mainz, Germany; fuermeyer@uni-mainz.de (F.F.); dmuenzbe@students.uni-mainz.de (D.M.); carrella@uni-mainz.de (L.M.C.)

* Correspondence: rentschler@uni-mainz.de; Tel.: +49-613-1392-5491

Table of content

1. NMR spectra
   ^{1}H-NMR of **L** p. 2
   ^{13}C-NMR of **L** p. 3
   Comparison of the ^{1}H-NMR-spectra of **C1** and **L** p. 4
2. Mass spectrum of **L** p. 5
3. IR spectra of dried **C1** and **C2** p. 6
4. X-ray diffraction measurements
   Molecular structure/asymmetric unit of **C1** at 173 K p. 7
   Molecular structure of **C2** at 120 K p. 7
   Molecular structure of **C2** at 250 K p. 8
   Crystallographic parameters for all structures of **C1** and **C2** p. 8

1. NMR spectroscopy

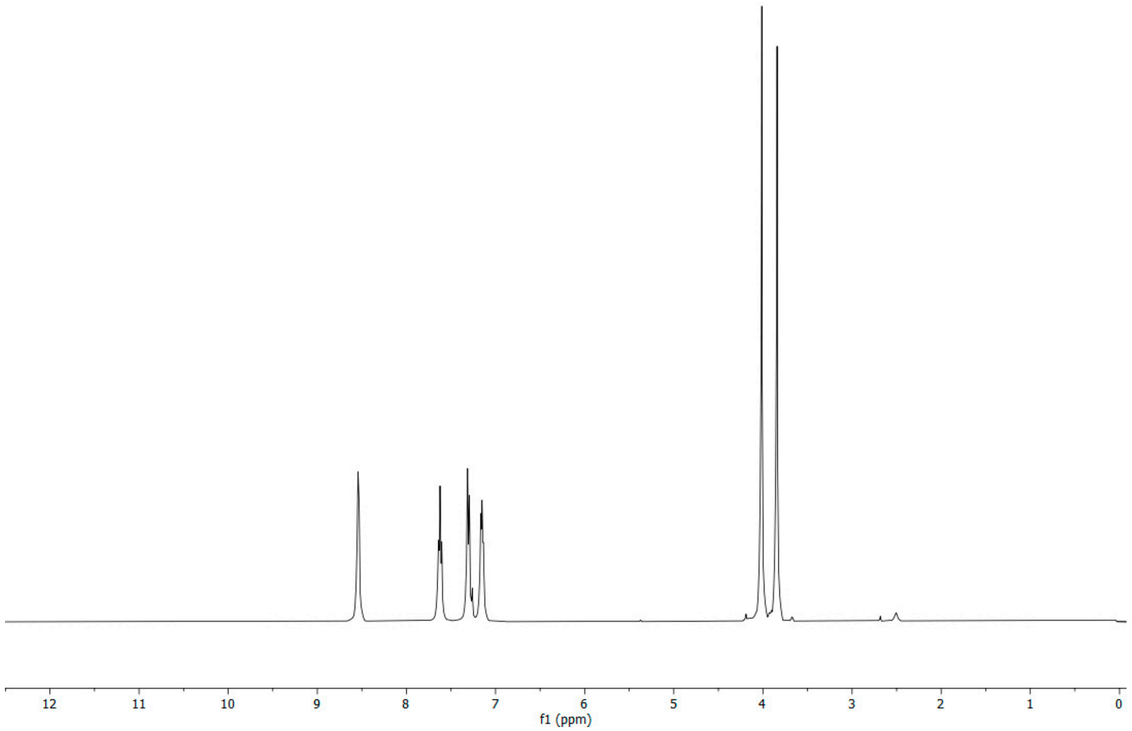

Figure S1. ^{1}H-NMR of 2,5-bis[(2-pyridylmethyl)thio]methyl-1,3,4-thiadiazole (L).

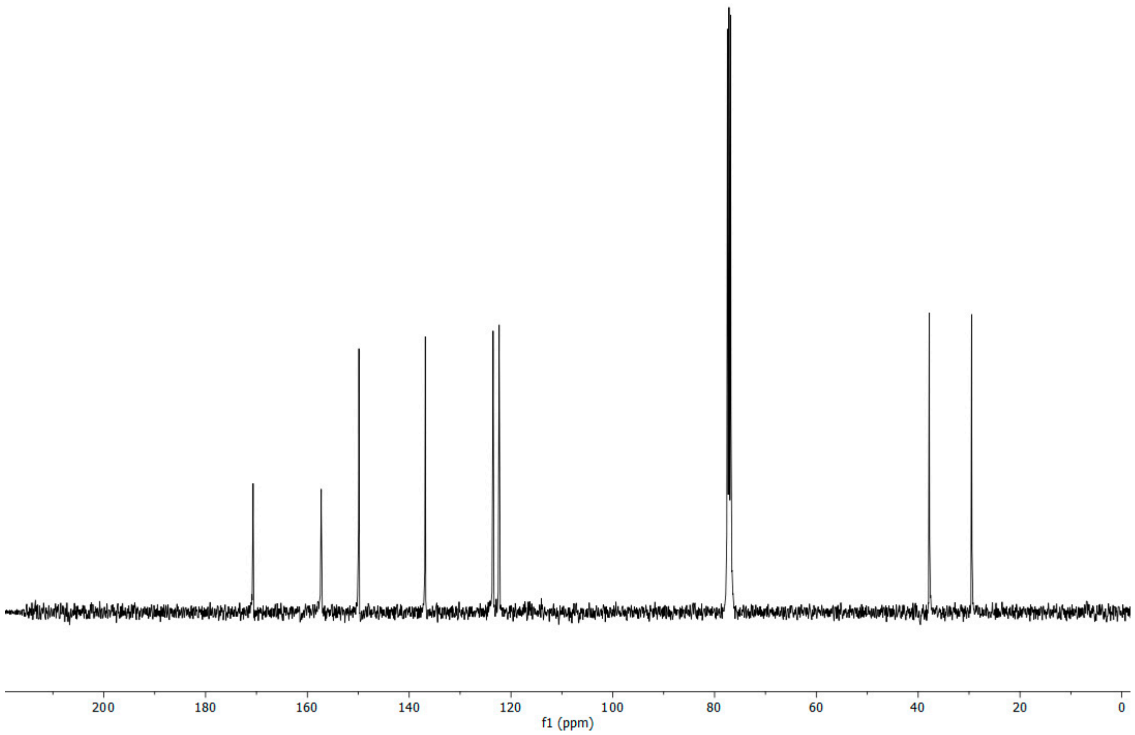

Figure S2. ^{13}C-NMR of 2,5-bis[(2-pyridylmethyl)thio]methyl-1,3,4-thiadiazole (L).

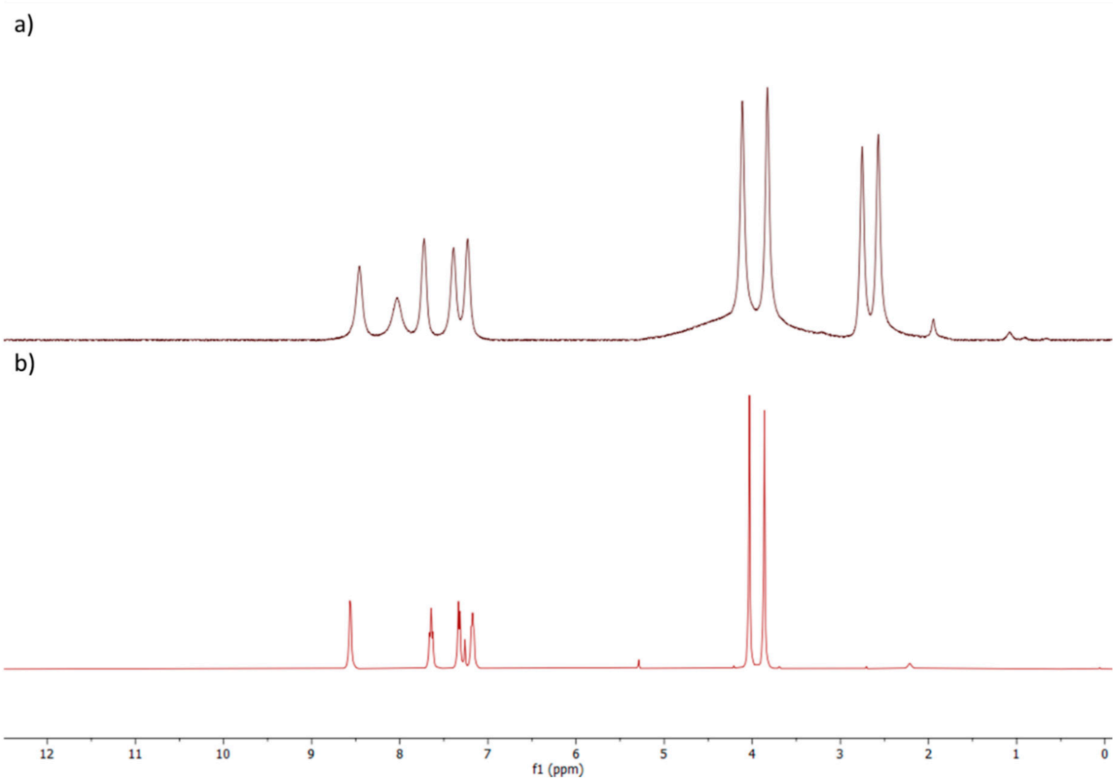

Figure S3. Comparison of the ^{1}H-NMR spectra of a) $[Fe^{II}(L)_2](ClO_4)_2$ (C1) in DMSO-d_6 and b) 2,5-bis[(2-pyridylmethyl)thio]methyl-1,3,4-thiadiazole (L) in $CDCl_3$.

2. Mass spectrometry

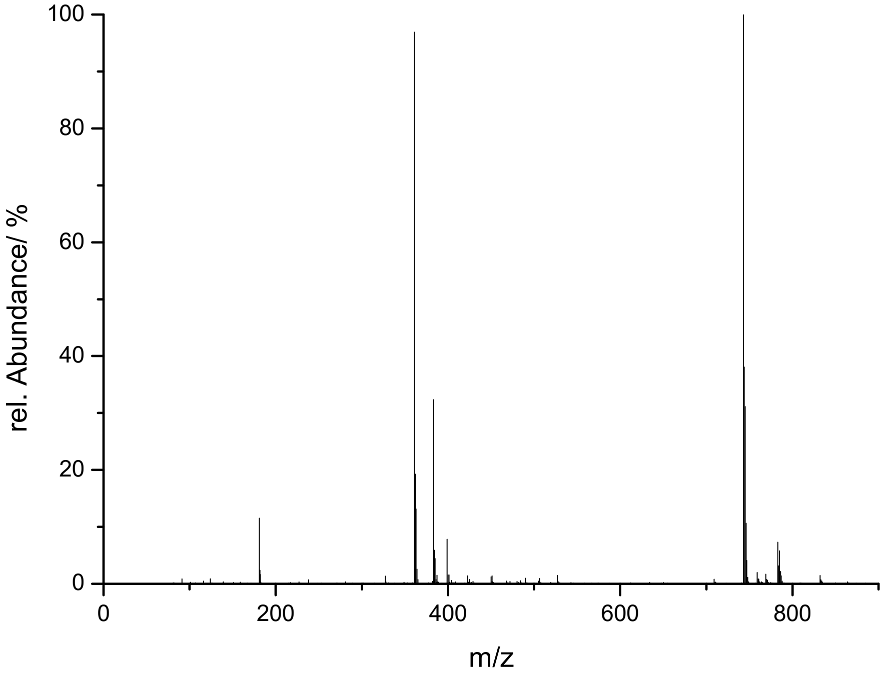

Figure S4. Mass spectrum of 2,5-bis[(2-pyridylmethyl)thio]methyl-1,3,4-thiadiazole (L).

3. IR spectroscopy

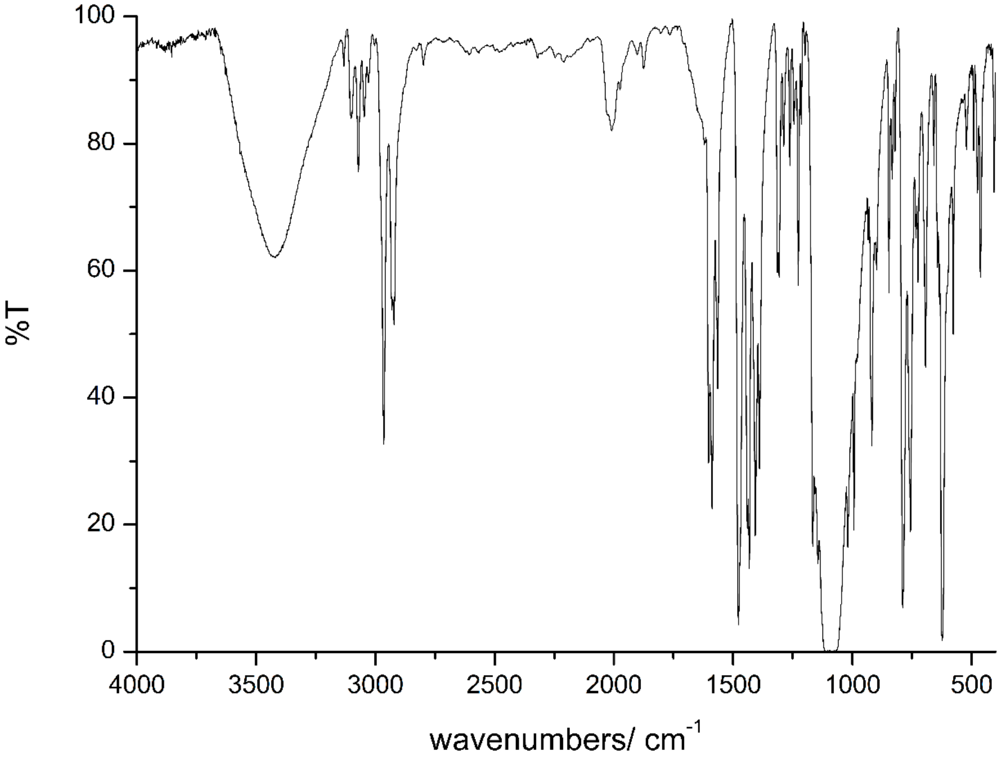

Figure S5. IR spectrum of dried $[Fe^{II}(L)_2](ClO_4)_2$ (C1).

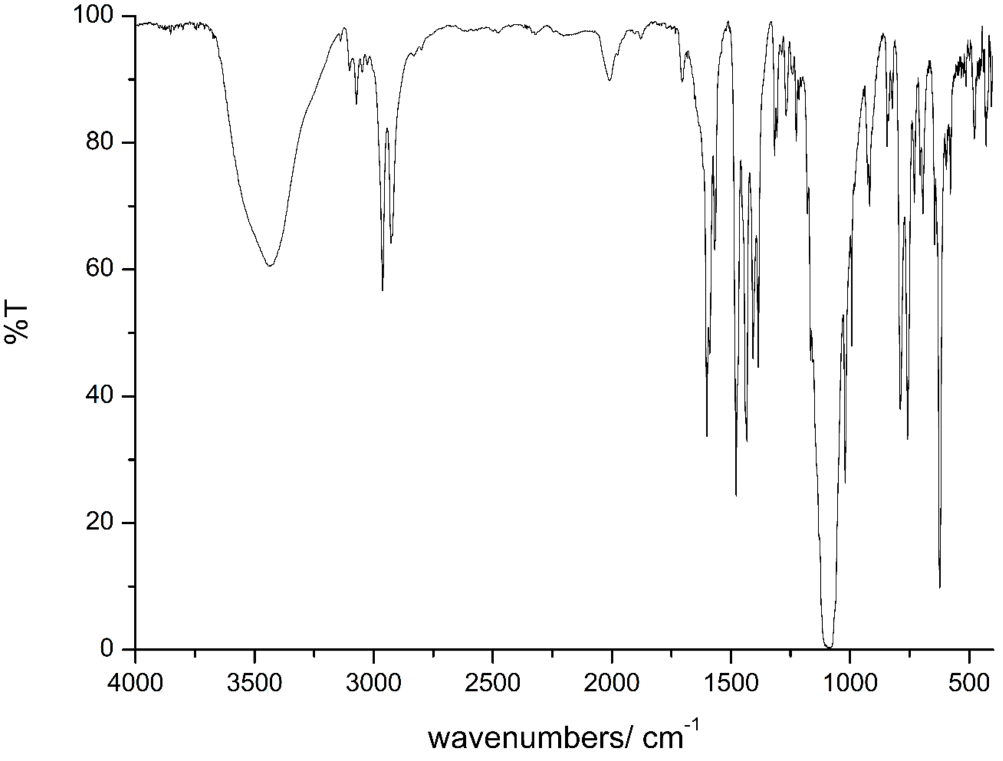

Figure S6. IR spectrum of dried $[Co^{II}(L)_2](ClO_4)_2$ (C2).

4. X-ray diffraction measurements

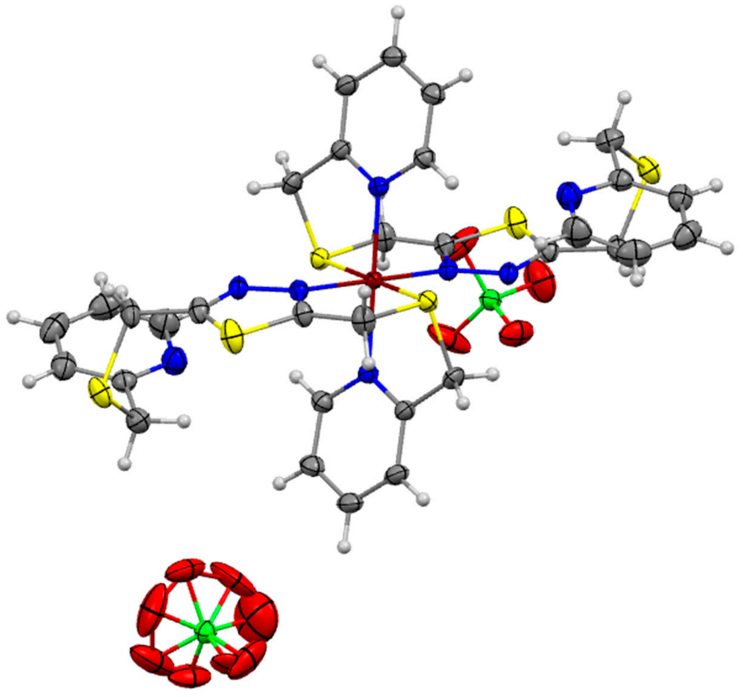

Figure S7. Molecular structure/asymmetric unit of $[Fe^{II}(L)_2](ClO_4)_2$ (C1) with thermal ellipsoids at 173 K. Color code: Fe dark red, N blue, S yellow, C grey, H light grey, Cl green and O red.

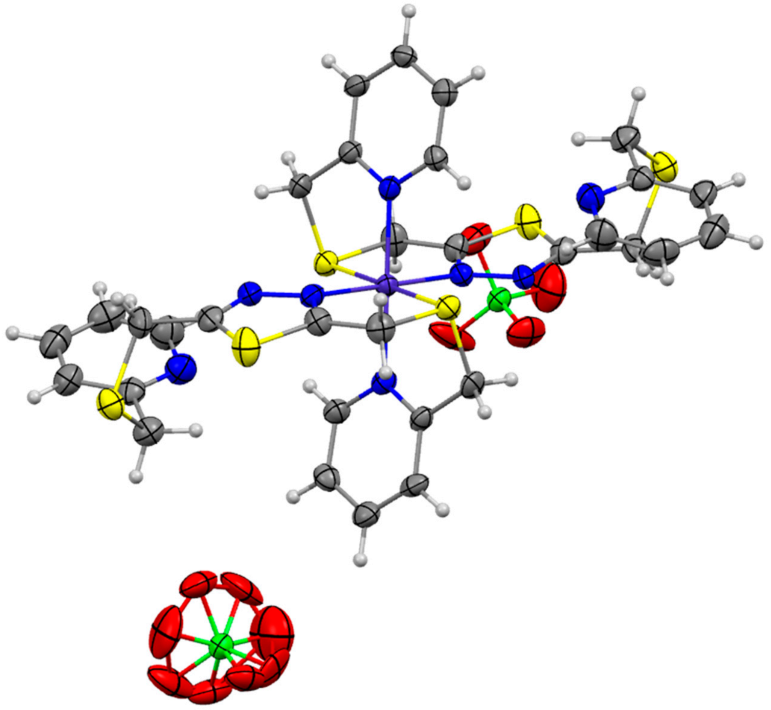

Figure S8. a) Molecular structure of $[Co^{II}(L)_2](ClO_4)_2$ (C2) with thermal ellipsoids at 120 K. Color code: Co dark blue, N blue, S yellow, C grey, H white, Cl green and O red.

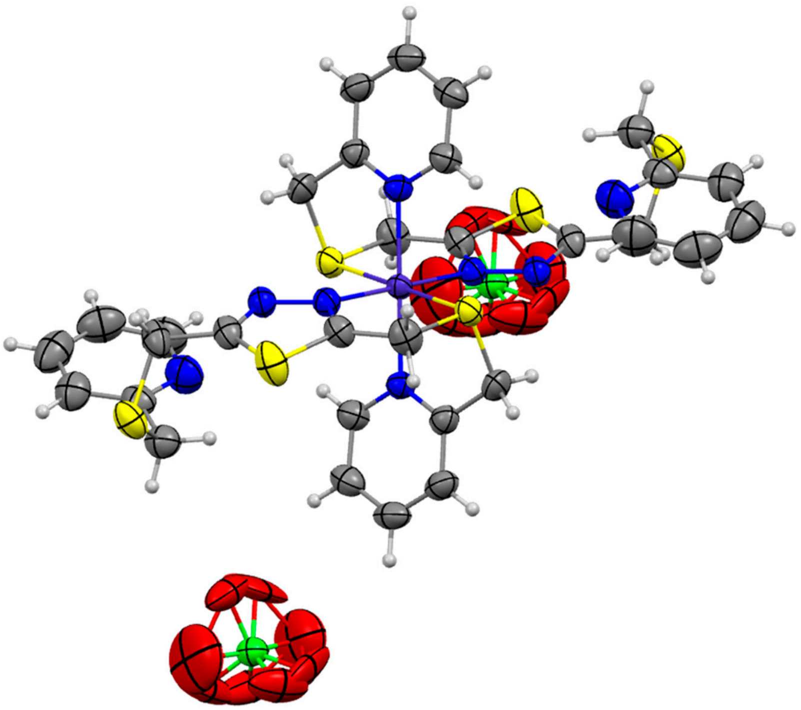

Figure S9. a) Molecular structure of $[Co^{II}(L)_2](ClO_4)_2$ (C2) with thermal ellipsoids at 250 K. Color code: Co dark blue, N blue, S yellow, C grey, H white, Cl green and O red.

Table S1. Crystallographic parameters for the discussed crystal structures of C1 and C2.

	**C1** (@173 K)	**C2** (@120 K)	**C2** (@250 k)
formula	C32 H32 Cl2 Fe N8 O8 S6	C32 H32 Cl2 Co N8 O8 S6	C32 H32 Cl2 Co N8 O8 S6
molar weigth [g/mol]	975.76	978.84	978.84
crystal system	monoclinic	monoclinic	monoclinic
space group	$P2_1/c$	$P2_1/c$	$P2_1/c$
a/Å	10.7228(2)	10.6468(8)	10.7440(13)
b/Å	18.2829(5)	18.1665(10)	18.254(2)
c/Å	20.0702(4)	20.2042(15)	10.9219(15)
α/°	90	90	90
β/°	97.117(2)	97.583(6)	111.706(10)
γ/°	90	90	90
V/Å^3	3904.3(2)	3873.6(5)	1990.24(4)
Z	4	4	2
T/K	173(2)	120(2)	250(2)
$\rho_{calcd.}$ [g/cm^3]	1.660	1.678	1.633
μ [mm^{-1}]	0.906	0.967	0.941
R(int)	0.0226	0.0487	0.0272
S	1.041	1.032	1.097
R1 (l > 2σ (l))	0.0365	0.1484	0.0944
wR2 (all data)	0.1033	0.1802	0.1148
